# A community effort for automatic detection of postictal generalized EEG suppression in epilepsy

**DOI:** 10.1186/s12911-020-01306-8

**Published:** 2020-12-24

**Authors:** Yejin Kim, Xiaoqian Jiang, Samden D. Lhatoo, Guo-Qiang Zhang, Shiqiang Tao, Licong Cui, Xiaojin Li, Robert D. Jolly, Luyao Chen, Michael Phan, Cung Ha, Marijane Detranaltes, Jiajie Zhang

**Affiliations:** 1 School of Biomedical Informatics, University of Texas Health Science Center at Houston, 7000 Fannin Street, 77030 Houston, TX USA; 2 Department of Neurology, McGovern Medical School, University of Texas Health Science Center at Houston, 7000 Fannin Street, 77030 Houston, TX USA

**Keywords:** Electroencephalographic, Postictal Generalized electroencephalographic suppression, Intermittent slow wave, Detection, Prediction, Physiological signal

## Abstract

Applying machine learning to healthcare sheds light on evidence-based decision making and has shown promises to improve healthcare by combining clinical knowledge and biomedical data. However, medicine and data science are not synchronized. Oftentimes, researchers with a strong data science background do not understand the clinical challenges, while on the other hand, physicians do not know the capacity and limitation of state-of-the-art machine learning methods. The difficulty boils down to the lack of a common interface between two highly intelligent communities due to the privacy concerns and the disciplinary gap. The School of Biomedical Informatics (SBMI) at UTHealth is a pilot in connecting both worlds to promote interdisciplinary research. Recently, the Center for Secure Artificial Intelligence For hEalthcare (SAFE) at SBMI is organizing a series of machine learning healthcare hackathons for real-world clinical challenges. We hosted our first Hackathon themed centered around Sudden Unexpected Death in Epilepsy and finding ways to recognize the warning signs. This community effort demonstrated that interdisciplinary discussion and productive competition has significantly increased the accuracy of warning sign detection compared to the previous work, and ultimately showing a potential of this hackathon as a platform to connect the two communities of data science and medicine.

## Introduction

Applying machine learning to healthcare sheds light on evidence-based decision making and has shown promises to improve healthcare by combining clinical knowledge and biomedical data. However, medicine and data science are not synchronized. Oftentimes, researchers with a strong data science background do not understand the clinical challenges, while on the other hand, physicians do not know the capacity and limitations of state-of-the-art machine learning methods. The difficulty boils down to the lack of a common interface between two highly intelligent communities due to the privacy concerns and the disciplinary gap. Data scientists have limited opportunities to access real healthcare data and many advanced machine learning models do not account for unique characteristics in clinical challenges. The lack of interpretability of black-box machine models can also reduce the enthusiasm for clinicians to apply them in practice. To address these challenges, we need to provide access to data and “formulate” clinical problems (often messy and complicated) in an informatics friendly way for algorithmic development. This is a critical mission for training the next generation of biomedical informaticians and accelerating healthcare research with machine learning. The School of Biomedical Informatics (SBMI) at UTHealth is a pilot in connecting both worlds to promote interdisciplinary research. Recently, the Center for Secure Artificial Intelligence For hEalthcare (SAFE) at SBMI is organizing a series of machine learning healthcare hackathons for real-world clinical challenges, which are carefully prepared for data science students/trainees to investigate and compete for best solutions with a dual mission for education and research. We developed a software platform “Interactive Data Analysis Research Ecosystem (IDARE)” to provide a secure platform with provisioned data and necessary computation resources to offer a unique opportunity for students/trainees to tackle emerging clinical challenges raised by physicians. Partnering with Texas Institute for Restorative Neurotechnologies (TIRN), we hosted our first Hackathon on September 24–25, 2019 themed centered around Sudden Unexpected Death in Epilepsy (SUDEP) and finding ways to recognize the warning signs. We incentivized smart young minds to join a 24-h Hackathon competition with the general sponsorship by Elimu Inc.

## Competition design

### Problem description

Epilepsy is a neurological disorder marked by sudden recurrent episodes of sensory disturbance, loss of consciousness, or convulsions, associated with abnormal electrical activity in the brain [1, 2]. Patients with epilepsy have sudden and unforeseen seizures regardless of the circumstance. Although it is rare, approximately 3000 people in the United States die every year from Sudden Unexpected Death in Epilepsy (SUDEP) because of a shutdown of brain, cardiac, and breathing functions. Prolonged postictal generalized electroencephalographic (EEG) suppression (PGES) appears to identify refractory epilepsy patients who are at risk of SUDEP. It has been reported that the relative risk of SUDEP is elevated with PGES duration of $>50$ s; the relative risk increases by 1.7% for each 1-s increase in the duration of PGES [3]. Since generalized tonic-clonic seizures (GTCS) are the most significant risk factor for SUDEP and PGES most often occurs after GTCS, PGES has been considered as a potential biomarker of SUDEP risk [3–5].

Determining the duration of PGES clinically has heavily relied on visual analysis of EEG signals, which requires extensive clinical experts’ manual review to annotate the end of PGES or the onset of the first intermittent slow-wave (ISW) activity, and sometimes shows inconsistent agreement between experts [6]. Therefore, it is highly desirable to develop automatic PGES detection tools to alleviate clinical experts’ manual efforts.

**Fig. 1:**
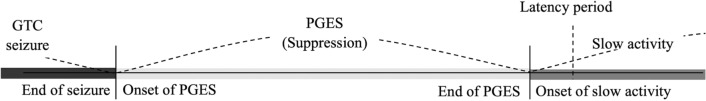
End of PGES and onset of first intermittent slow activity. Our objective is to detect the transition of PGES to the slow activity during the latency period. *GTC* generalized tonic-clonic, *PGES* postictal generalized electroencephalographic suppression

Signal processing and machine learning have been extensively used for epileptic seizure detection [7]. They are based on extracting features from time domain, frequency domain, or wavelet (time and frequency) domain together with classification algorithms. The time domain features include variance, skewness, and kurtosis [8]; the frequency domain features include energy or amplitude (peak frequency, median frequency) [9]; the wavelet domain features include spectrogram. These extracted features are then fed into various classification algorithms such as a k-nearest neighbor, support vector machines, or random forest. Recently convolutional neural networks have been used with raw EEG signals [10]. While epileptic seizure detection using EEG has been widely studied, there has been little attempt to develop automatic PGES detection models [6, 11]. A critical challenge of applying machine learning approaches to detect the end of PGES is that EEG signals may be noisy due to multiple potential sources of artifacts, such as eye movement, breathing, and muscle artifacts. To tackle this challenge, we organized a 24-h-long Hackathon as a community effort to develop innovative algorithms to detect the end of PGES. The objective of this Hackathon was to build machine learning models to detect the transiting point from the offset of PGES to the onset of the first ISW within a predefined latency period (no later than 10 s after actual onset) (Fig. 1).

### Patient cohort

We analyzed 5-min-long 168 EEG signals after GTCS, collected from TIRN. The clinical annotation of the end of suppression was obtained by clinical experts. The patient’s demographic information is described in Table 1. We split the PGES patients into 80% training (n = 134) and 20% test (n = 34).

**Table 1:** Patient’s demographic information

Mean age at onset of epilepsy, year	20.043
*Sex*	
Female	71
Male	57
*Etiology*	
Idiopathic	87
Cryptogenic	3
Hippocampal sclerosis	10
Cortical dysplasia	6
Post encephalitis	3
Post traumatic	3
Tumor	9
Vascular malformations	3
Cerebral infarction	2
Structural	2
*Seizure type*	
Complex partial	2
Complex partial—generalized tonic–clonic	98
Complex partial—generalized clonic	2
Primary generalized tonic–clonic	22
Myoclonic seizure	2
*Epilepsy syndrome*	
Left temporal lobe	34
Right temporal lobe	17
Bitemporal lobe	10
Left frontal lobe	8
Right frontal lobe	5
Frontal lobe (non-lateralizable)	5
Left occipital epilepsy	1
Right occipital epilepsy	0
Left hemisphere	14
Right hemisphere	11
Idiopathic generalized	24
*MRI*	
Lesion positive	62
Lesion negative	51

### EEG signal preparation

The EEGs were sampled from 13 electrodes that capture temporal and spatial patterns of the brain. We used 10 standard bipolar EEG montages (pairwise offsets of two adjacent electrodes): Fp1-F7; F7-T7; T7-P7; P7-O1; Fp2-F8; F8-T8; T8-P8; P8-O2; Fz-Cz; and Cz-Pz from the 13 electrodes (Fp1, Fp2, O1, O2, F7, F8, T7, T8, P7, P8, Fz, Cz, Pz). We aligned the various sampling rates (ranging from 150 to 256 Hz) to 200 Hz.

### Submission

Submissions were judged on the accuracy of detection—area under the receiver operating curve (AUC). Participants were asked to identify whether given short segments of EEG signals (i.e., clips) contain the onset of slow activity or not. The slow activity clip did not include slow activity signals beyond 10 s after onset.

### Baseline model

**Fig. 2:**
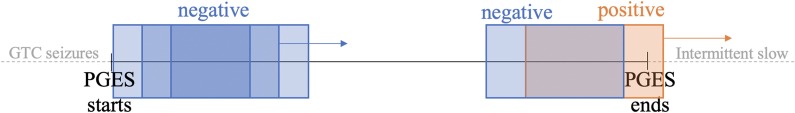
Data augmentation. If the crop reaches the end of PGES, then the crop was set as positive (i.e., label = 1), otherwise negative (i.e., label = 0)

The organizers developed a baseline model to assess the difficulty of the hackathon problem and guide participants to avoid pitfall using the organizer’s trial-and-error. The organizer’s baseline model was based on augmenting the sequence via cropping and applying a deep learning method. The baseline model cropped one EEG recording during PGES into multiple crops to boost the training sample size from the limited number of subjects (Fig. 2). We adopted the cropping strategy from object recognition in images and movement-related EEG signals [12, 13]. We set a sliding time window of fixed length with a cropping stride. We assume that real-time detection should be made no later than a certain latency period after PGES ends. Per-crop labels were positive if the crops reach or pass the end of PGES; negative if the crops lie in PGES. After cropping, we have a total of 296,188 crops—240,511 for training (80%) and 55,677 for test (20%). After performing threshold analysis for the length of time window, stride, and detection latency period, we set them 10 s, 100 ms, and 10 s, respectively. That is, all detection was based on the 10 s time window without seeing the future (no retrospective review).

**Fig. 3:**
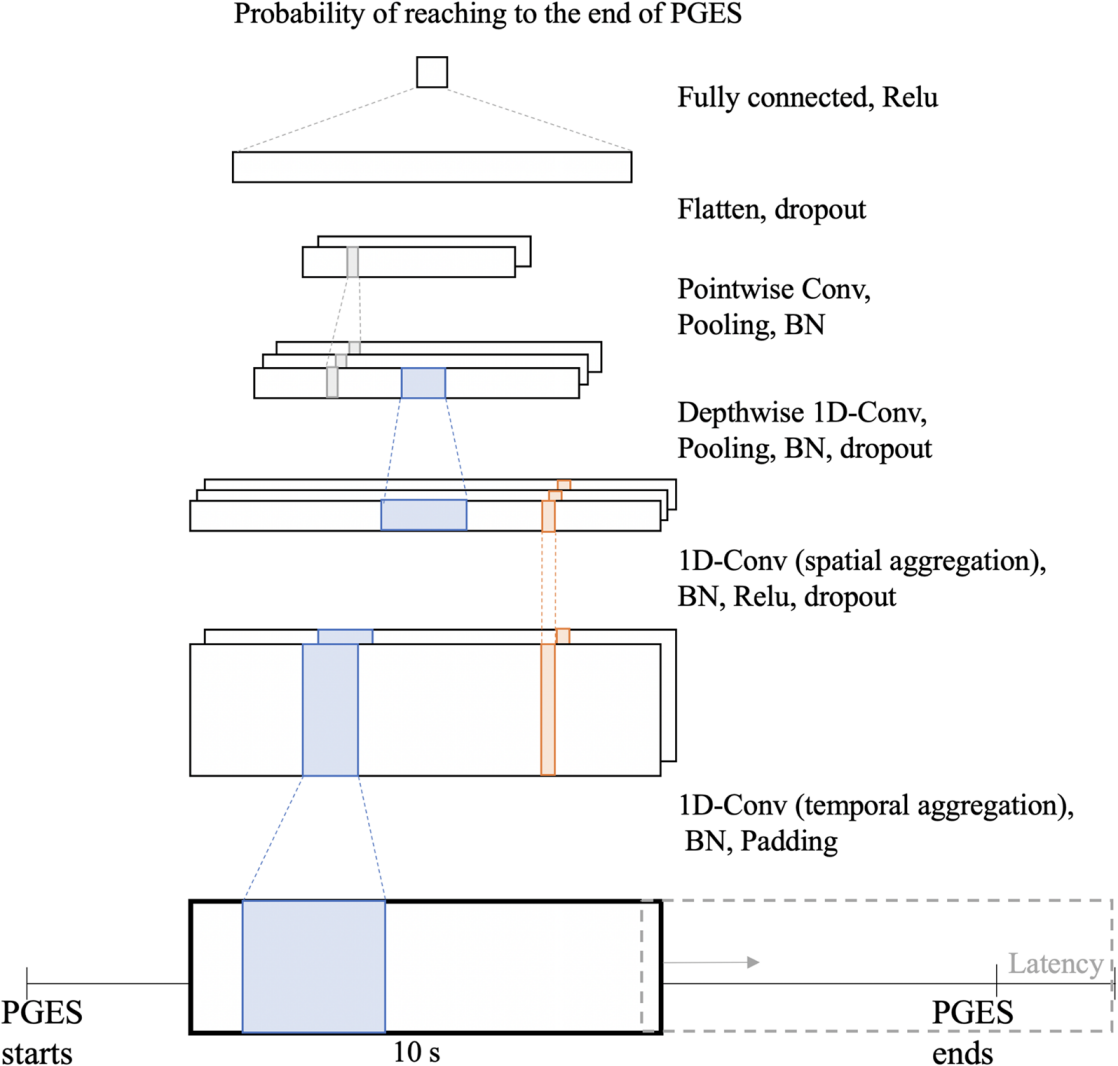
CNN-based classifier for real-time suppression detection. Input were raw EEG segments cropped from sliding windows during PGES and latency periods. Output was the probability that PGES ends

**Fig. 4:**
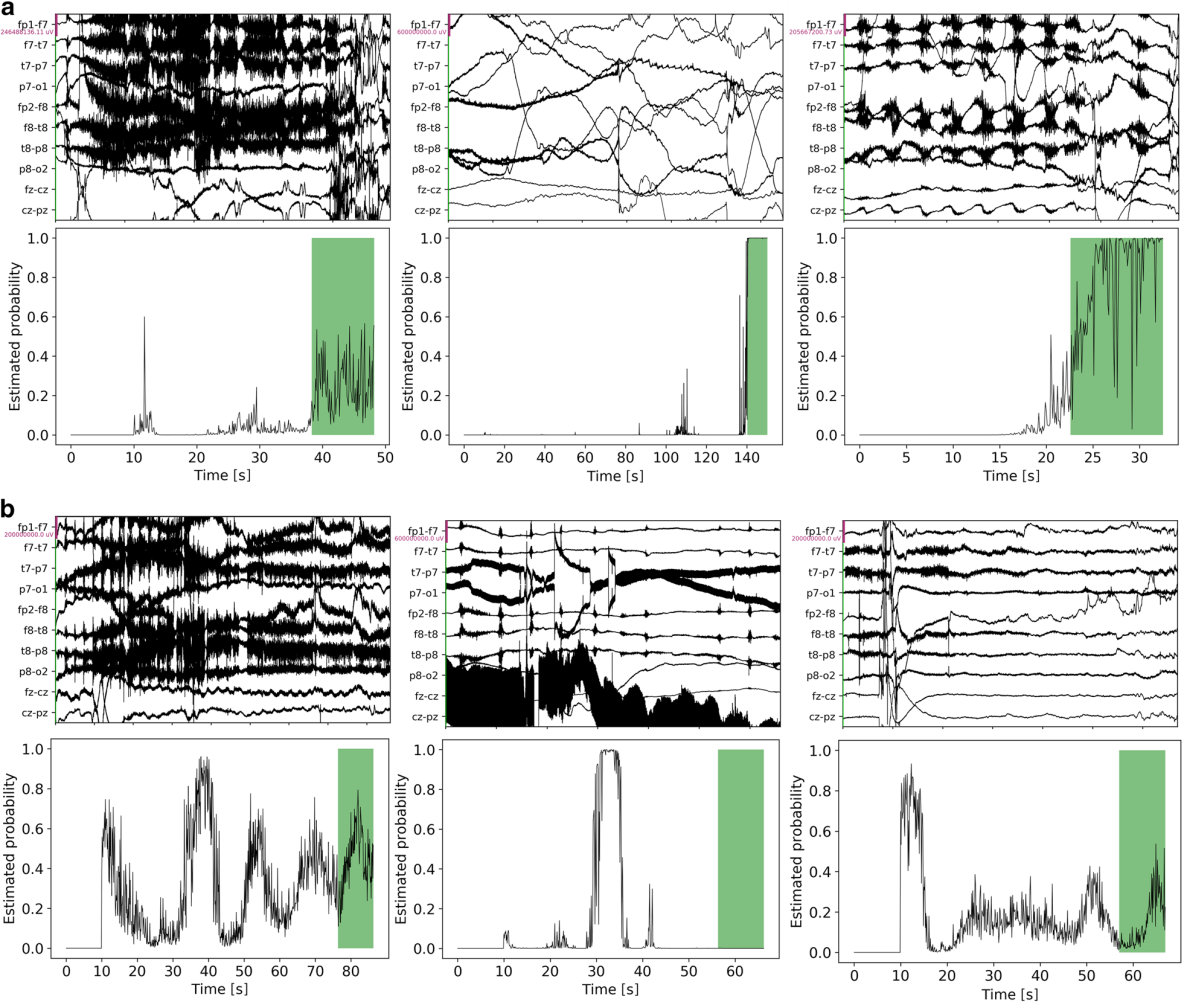
Comparing raw EEG signals and the estimated probability of slow activity. The green area refers to slow activity. **a** True positive detection. **b** False positive or false negative detection

Once we extracted the crops, we formulated the detection of the end of suppression as a binary classification task in which the model classifies whether the current time window crop reaches the end of suppression. As one of non-linear classifiers, we designed a customized convolutional neural network (CNN) for EEG signals, inspired by EEGnet [14]. The proposed model integrated feature extraction and classification in an end-to-end manner, which allows us to avoid time-intensive feature engineering of EEG signals. It encoded the temporal trends and spatial trends at a time (Fig. 3). The first layer was 1-dimensional convolution (with a filter size of 18) to convolute and aggregate temporality of raw EEG signals. These multiple temporal filters can implicitly learn the intensity of different frequency bands. The second layer was a 1-dimensional convolutional layer (with a filter size of 101) for spatial aggregation across different montages in the scalp. This convolution can capture distinct activation in different scalp areas with different frequency bands. Then we applied depthwise temporal convolution and pointwise convolution to aggregate spatio-temporal features and in turn reduce the feature size. The final layer was a fully-connected one with flattened features. We applied batch normalization (BN), Relu non-linear activation, and dropout between these convolutional layers. Training inputs were the fixed-length crops and label per crop was a binary indicator whether the crops reach the end of PGES. The loss function was binary cross-entropy and the optimizer was Adam implemented in Pytorch. Our proposed model continuously detected the end of PGES at every 100 ms. The proposed model achieved AUC of 0.77 within detection latency of 10 s. We visualized the estimated probability computed from the proposed model and compared it with the actual onset time of intermittent slow (Fig 4). We observed that for some cases the estimated probability is aligned with the raw EEG signals (Fig. 4a); whereas in other cases, the estimated probability does not hit the right onset time (Fig. 4b).

## Competition results

We received 42 registrations from five universities in the greater Houston area (Rice University, Texas A&M University, University of Houston, Prairie View A&M University, and University of Texas Health Science Center at Houston). Among them, 12 contestants submitted their final results during the 24 h. In total there were 88 submissions from the 12 contestants. Finally, Lamichhane from Rice University won the competition. In addition, three contestants extended their work and published them in this BMC Medical Informatics and Decision Making special issue. The details of the performance are summarized in Table 2. Lamichhane et al. derived 127 features from time or frequency features (correlation, the temporal signal ratio in sliding windows) used a random-forest based classification framework to detect the end of PGES. The features used captured both the inter-channel dynamics, e.g. with correlation features and intra-channel dynamics, e.g. by comparing the temporal ratio of signal properties. The authors obtained an AUC of 0.84 in the final classification evaluation using the test set. This accuracy was significantly higher than the previous work [6, 11] and organizer’s baseline model. Vance et al. combined a pre-activation style residual neural network with regularization and sampling strategies to train a model that can effectively generalize with a limited amount of training data. The experimental results show that the described method is significantly more accurate than the naive baseline when applying deep residual networks to the problem. Zhu et al. proposed a convolutional neural network with light architecture for slow activity prediction. The model also explored the impact of random noise of EEG signal in the model’s performance by applying denoising filters. It took about 20 s to train the model using a batch size of 64 samples with 10 s signals and 10 montages.

**Table 2:** Top contestant’s methods and innovations

Methods	AUC	Reference
Time and frequency features, Random forest	0.83	Lamichhane (Rice University)
ResNet, visualization	0.77	Vance (University of Houston)
Hann filtering, lightweight CNN	0.72	Zhu (University of Texas Health Science Center)

Mier et al. proposed an architecture that includes augmenting the data set using an EEG specific feature extraction process (pyEEG) and implementing a classification approach using Gradient Boosted Decision Trees. Feature calculations include SVD Entropy, Petrosian Fractal Dimension, and Power Spectral Intensity, which were the highest performers.

The algorithms developed in this Hackathon demonstrated the potential of the automatic detection of PGES. Various features from time and domain and a mixture of them won the competition. Convolutional neural network approaches showed comparable accuracy without extensive feature engineering. The lightweight CNN also showed potentials in efficiency for real-world deployment in clinical settings. In this collaborative community effort, we have demonstrated that interdisciplinary discussion and productive competition has significantly increased the PGES detection accuracy compared to the previous work and organizer’s baseline. In addition, various contestants provided various perspectives on supporting clinician’s manual monitoring activity using such denoising and visualization. Ultimately we have found the potential of this hackathon as a platform to connect the two communities of data science and medicine.

## Data Availability

The data include protected health information, thus are not publicly available.

## Abbreviations

SUDEP: Sudden unexpected death in epilepsy
IDARE: Interactive data analysis research ecosystem
EEG: Electroencephalographic
PGES: Prolonged postictal generalized electroencephalographic suppression
ISW: Intermittent slow wave
GTCS: Generalized tonic–clonic seizures
AUC: Area under the receiver operating curve
CNN: Convolutional neural network
BN: Batch normalization
